# Clinical and serological predictors of relapse in pemphigus: a study of 143 patients

**DOI:** 10.1111/ced.14854

**Published:** 2021-08-27

**Authors:** G. Genovese, C. A. Maronese, G. Casazza, L. Corti, L. Venegoni, S. Muratori, E. Berti, D. Fanoni, A. V. Marzano

**Affiliations:** ^1^ Dermatology Unit Fondazione IRCCS Ca' Granda Ospedale Maggiore Policlinico Milan Italy; ^2^ Department of Pathophysiology and Transplantation Università degli Studi di Milano Milan Italy; ^3^ Department of Biomedical and Clinical Sciences ‘L. Sacco’ Università degli Studi di Milano Milan Italy

## Abstract

**Background:**

Pemphigus is an autoimmune bullous disease mediated by autoantibodies targeting epithelial cell–cell adhesion molecules. Predictors of relapse have not yet been clearly identified.

**Aims:**

To identify factors at diagnosis and during follow‐up that could be predictors of relapse.

**Methods:**

Clinical and immunopathological data at diagnosis, clinical remission and first relapse from patients with pemphigus vulgaris or foliaceus and at least a 36‐month follow‐up were collected retrospectively. Based on the autoantibody profile at diagnosis, three serological patient subsets were devised: (i) anti‐desmoglein (Dsg)1‐positive and anti‐Dsg3‐negative; (iii) anti‐Dsg1‐negative and anti‐Dsg3‐positive; and (iii) anti‐Dsg1‐positive and anti‐Dsg3‐positive.

**Results:**

Data from 143 patients were collected. No significant differences were found between relapsers (*n* = 90) and nonrelapsers (*n* = 53) for time to remission or for anti‐Dsg1 and anti‐Dsg3 titres at diagnosis and remission. In the analysis of all patients, a higher risk of relapse was found for a body surface area (BSA) score of 3 compared with BSA < 3 (OR = 3.30, 95% CI 1.17–9.28; *P* = 0.02) and for a positive titre of either anti‐Dsg1 or anti‐Dsg3 autoantibodies at remission compared with both being negative (OR = 2.42, 95% CI 1.21–4.85, *P* = 0.01). In patients who were anti‐Dsg3‐positive and anti‐Dsg1‐negative at diagnosis, failure to achieve anti‐Dsg3 negativity at clinical remission was a significant predictor of relapse (OR = 7.89, 95% CI 2.06–30.21; *P* < 0.01). Similarly, failure to achieve anti‐Dsg1 negativity at clinical remission was a significant predictor of relapse in patients with both anti‐Dsg1 and anti‐Dsg3 positivity at diagnosis (OR = 5.74, 95% CI 1.15–28.61; *P* = 0.03), but not in those who were anti‐Dsg1‐positive/anti‐Dsg3‐negative at diagnosis (OR = 1.08, 95% CI 0.27–4.30; *P* = 0.91).

**Conclusion:**

Regardless of pemphigus subtype, autoantibody titre negativity at clinical remission in patients classified based on their anti‐Dsg1 and anti‐Dsg3 profile at diagnosis and BSA were useful tools in predicting relapse.

## Introduction

Pemphigus comprises a group of mucocutaneous autoimmune bullous diseases mediated by circulating autoantibodies targeting epithelial cell–cell adhesion molecules of the cadherin family, particularly desmoglein (Dsg)1 and Dsg3.^1^ Most of these autoantibodies may be detected and quantified by means of ELISA analysis.^1^ The most frequent variant of pemphigus is pemphigus vulgaris (PV), which typically exhibits a chronic relapsing course and is characterized by most relapses occurring within 2 years of diagnosis.^2^ A less frequent variant is pemphigus foliaceus (PF), which is generally considered more responsive to treatment and is rarely characterized by a chronic course.^3^

Although many studies have explored the role of different clinical and immunopathological factors in predicting relapse, markers predictive of relapse have not yet been clearly identified. The presence of mucosal involvement at pemphigus onset^4^, ^5^ and the positivity of direct immunofluorescence in patients with PV in clinical remission^6^ have been found to be associated with a higher risk of relapse. Ujiie *et al*.^7^ also showed that in patients with mucocutaneous PV, initial doses of systemic corticosteroids were significantly lower in relapsing than in nonrelapsing cases.

Changes in titres of circulating autoantibodies evaluated by means of ELISA anaysis may help the clinician undertake therapeutic decisions in the remission phase.^8^ A correlation between anti‐Dsg1 and anti‐Dsg3 titres and disease activity has been widely demonstrated,^9^, ^10^, ^11^, ^12^, ^13^, ^14^, ^15^, ^16^ even though serial ELISAs cannot be considered absolute indicators of disease activity. Indeed, elevated autoantibody titres may persist in phases of clinical remission,^17^, ^18^ conceivably due to a high percentage of nonpathogenetic autoantibodies (e.g. IgG1‐type autoantibodies or autoantibodies directed against nondisease‐associated epitopes) in these patients. The correlation of anti‐Dsg1 antibodies with skin relapses would seem to be more significant than that of anti‐Dsg3 antibodies with mucosal relapses.^9^, ^14^, ^19^ However, Daneshpazhooh *et al*.^6^ demonstrated that positive titres (> 20 U/mL) of anti‐Dsg3 were associated with earlier relapse in patients with PV in remission. In a prospective study on pemphigus relapses in rituximab‐treated patients, lower Dsg1 levels were associated with longer time to relapse.^20^ Finally, another study on biomarkers predictive of relapse in rituximab‐treated pemphigus patients showed that the relapse was associated with positivity for either anti‐Dsg1 or anti‐Dsg3 antibodies in serial ELISA tests after rituximab treatment.^21^

The primary endpoints of this single‐centre study were (i) to compare the demographic, clinical and immunopathological features at diagnosis and during follow‐up between relapsing and nonrelapsing patients, and (ii) to identify factors at diagnosis and during follow‐up that could be predictive of relapse.

## Methods

Because of the retrospective nature of the study, only a notification to the ethics committee of the Fondazione IRCCS Ca' Granda Ospedale Maggiore Policlinico (Milan, Italy) was requested. The study was conducted in accordance with the Declaration of Helsinki and all patients provided written informed consent for study participation.

### Patients

The clinical data of all patients with pemphigus seen in the 2007–2019 period at the Dermatology Unit of the Fondazione IRCCS Ca' Granda Ospedale Maggiore Policlinico of Milan were retrospectively analysed.

Eligibility criteria were (i) diagnosis of PV or PF based on typical findings on clinical (mucosal and/or cutaneous blisters and/or erosions), histopathological (acantholysis) and immunopathological (IgG and/or C3 intercellular deposits on direct and/or indirect immunofluorescence microscopy) examinations; (ii) positivity at onset of at least one anti‐Dsg1 or anti‐Dsg3 test; (iii) availability of clinical data and of anti‐Dsg1 and anti‐Dsg3 ELISAs at diagnosis, clinical remission and first relapse; and (iv) a follow‐up period of at least 36 months from diagnosis. Patients who did not achieve clinical remission during the follow‐up period, patients with pemphigus subtypes other than PV or PF, and patients treated with rituximab before the first relapse were excluded.

Four clinical phenotypes^22^ were distinguished: (i) cutaneous (c)PV (characterized by suprabasal acantholysis, presence of anti‐Dsg3 autoantibodies with or without anti‐Dsg1 antibodies and exclusively cutaneous lesions^23^); (ii) mucosal (m)PV (suprabasal acantholysis, presence of anti‐Dsg3 antibodies with or without anti‐Dsg1 antibodies and exclusively mucosal lesions); (iii) mucocutaneous (mc)PV (suprabasal acantholysis, presence of anti‐Dsg3 antibodies with or without anti‐Dsg1 antibodies and lesions of the skin and mucous membranes); and (iv) PF (superficial acantholysis, presence of anti‐Dsg1 autoantibodies without anti‐Dsg3 autoantibodies and no mucosal involvement).

### Clinical and serological assessment: definitions

Based on criteria adapted from the consensus‐based definitions proposed by Murrell *et al*.,^24^ clinical remission was defined as the absence of old or new lesions for at least 2 months in a patient with minimal (prednisone or equivalent at a dosage of < 10 mg/day and/or minimal adjuvant therapy) or no therapy, while relapse was defined as the onset of > 3 new lesions (blisters, erosions) per month that do not heal within 1 week, or the extension of established lesions in a patient who had achieved clinical remission.

To avoid potential misclassification, a patient was considered negative for anti‐Dsg1 and/or anti‐Dsg3 autoantibodies at clinical remission only if no subsequent positivity was recorded in the following 6 months. Similarly, a patient was considered as positive for anti‐Dsg1 and/or anti‐Dsg3 autoantibodies at clinical remission only if no subsequent negativity was recorded in the following 6 months. Further serological changes occurring > 6 months after the achievement of clinical remission were not taken into consideration in those who did not relapse during the available follow‐up period.

Similarly to the report of Ujiie *et al*.^7^ skin severity at diagnosis was graded according to involved body surface area (BSA) [0 (no lesions), 1 (up to 5% of BSA involved), 2 (5–15% of BSA involved), 3 (> 15% of BSA involved)] and oral severity was graded according to oral cavity surface area involved (OSA) [0 (no lesions), 1 (up to 5% of OSA involved), 2 (5–30% of OSA involved), 3 (>30% of OSA involved)].^7^ BSA and OSA scores were always documented at each visit by the same investigators (GG and AVM) and were available in their written reports. In all cases, total body clinical photographs were also available and were used for confirmation.

The following data were collected: sex; age at onset; pemphigus subtype; mucosal and/or cutaneous involvement; involvement of oral, nasal, laryngeal, conjunctival, anogenital and mucosal locations; BSA; OSA; anti‐Dsg1 and anti‐Dsg3 autoantibody titre at diagnosis, remission and first relapse; time from diagnosis to clinical remission and to first relapse; treatment at diagnosis and at first relapse; and duration of follow‐up.

Based on anti‐Dsg1 and anti‐Dsg3 autoantibody profile at diagnosis, three serological subsets were created: (i) anti‐Dsg1‐positive and anti‐Dsg3‐negative; (ii) anti‐Dsg1‐negative and anti‐Dsg3‐positive; and (iii) anti‐Dsg1‐positive and anti‐Dsg3‐positive.

### ELISA test

To identify anti‐Dsg1 and anti‐Dsg3 autoantibodies in patient serum, an ELISA test (MESACUP Desmoglein‐1 and MESACUP Desmoglein‐3 respectively; MBL, Nagoya, Japan) was used, in accordance with the manufacturer's instructions. A cut‐off value of > 20 U/mL was used for both type of test.

### Statistical analysis

Categorical data are reported as *n* (%), whereas continuous variables are reported as median [interquartile range IQR)]. Comparisons of clinical and serological features between relapsing and nonrelapsing patients were performed using Fisher exact test (for categorical variables) or Mann–Whitney nonparametric test (for continuous variables). Correlation between quantitative variables was assessed with Spearman rank correlation coefficient (ρ).

Considering only relapsing patients, variation of anti‐Dsg1 and anti‐Dsg3 from diagnosis to first relapse was calculated for each patient (within‐patient analysis), then the nonparametric sign test for paired data was used to investigate whether anti‐Dsg1 and anti‐Dsg3 values changed from diagnosis to relapse. Subgroup analyses were also conducted considering pemphigus subtypes. Finally, univariate and multivariate logistic regression analyses were performed to assess the effect of some predefined factors on the risk of relapse. The following factors were considered as potential predictors of relapse in univariate models: age, sex, pemphigus subtype, mucosal and cutaneous involvement, BSA, OSA, nasal and laryngeal involvement, anogenital involvement, ocular involvement, anti‐Dsg1 and anti‐Dsg3 positivity at diagnosis, anti‐Dsg1 and anti‐Dsg3 negativity at remission (at least one of the two or both). Only those factors that showed a statistically significant association at univariate stage were considered in the multivariate model.

Logistic regression analyses were carried out on the whole sample and on the three subgroups identified according to anti‐Dsg1 and anti‐Dsg3 autoantibody profile at diagnosis, as described above: (i) anti‐Dsg1‐positive and anti‐Dsg3‐negative; (ii) anti‐Dsg1‐negative and anti‐Dsg3‐positive; and (iii) anti‐Dsg1‐positive and anti‐Dsg3‐positive.

Odds ratio and 95% CI were obtained from logistic models. All statistical analyses were conducted with the statistical software SAS (V9.4; SAS Institute, Inc., Cary, NC, USA), and two‐sided *P* values of < 0.05 were considered statistically significant.

## Results

### Demographic, clinical and immunopathological features of the whole patient cohort

As shown in Table 1, data were collected on 143 patients with pemphigus [83 (58.0%) women 60 (42.0%); men], with a median age at onset of 55 years (IQR 43–67 years). Of these, 29 (20.3%) patients were identified with PF, 15 (10.5%) with cPV, 27 (18.9%) with mPV and 72 (50.4%) with mcPV (Table S1). Of the 143 patients, 90 (62.9%) had experienced at least one relapse, whereas the remaining 53 (37.1%) had never experienced relapses. Median follow‐up time was 74 months (IQR 58–98 months).

**Table 1:** Clinical and serological features of relapsing and nonrelapsing patients with pemphigus.

Parameter	Patients		
	Relapsing (*n* = 90)	Nonrelapsing *n* = 53)	All (*n* = 143)
Males, *n* (%)	35 (38.9)	25 (47.2)	60 (42.0)
Age at onset, years; median (IQR)	54 (42–67)	56 (46–66)	55 (43–67)
Pemphigus subtype at diagnosis, *n* (%)			
PF	20 (22.2)	9 (17.0)	29 (20.3)
cPV	10 (11.1)	5 (9.4)	15 (10.5)
mPV	19 (21.1)	8 (15.01)	27 (18.9)
mcPV	41 (45.6)	31 (58.5)	72 (50.3)
Involved mucosal sites, *n* (%)			
Oral mucosa	58 (64.4)	37 (69.8)	95 (66.4)
Nasal and laryngeal mucosa	12 (13.3)	7 (13.2)	19 (13.3)
Anogenital mucosa	12 (13.3)	7 (13.2)	19 (13.3)
Conjunctiva	6 (6.7)	3 (5.7)	9 (6.3)
BSA, *n* (%)			
0	18 (20.0)	8 (15.1)	26 (18.2)
1	14 (15.6)	13 (24.5)	27 (18.9)
2	35 (38.9)	27 (50.9)	62 (43.4)
3	23 (25.6)	5 (9.4)	28 (19.6)
OSA, *n* (%)			
0	32 (35.6)	16 (30.2)	48 (33.6)
1	12 (13.3)	5 (9.4)	17 (11.9)
2	32 (35.6)	26 (49.1)	58 (40.6)
3	14 (15.6)	6 (11.3)	20 (14.0)
Therapy at diagnosis, *n* (%)			
Systemic corticosteroid plus immunosuppressive adjuvant therapy	50 (55.5)	24 (45.3)	74 (51.7)
Systemic corticosteroid monotherapy	40 (44.4)	29 (54.7)	69 (48.3)
Immunosuppressive monotherapy	0	0	0
Prednisone equivalent dose (mg/kg/day); mean ± SD	1.21 ± 0.80	1.12 ± 0.77	1.17 ± 0.79
Therapy at relapse, *n* (%)			
Systemic corticosteroid plus immunosuppressive adjuvant therapy	36 (40.0)	–	–
Systemic corticosteroid monotherapy	42 (46.7)	–	–
Immunosuppressive monotherapy	1 (1.1)	–	–
None	11 (12.2)	–	–
Prednisone equivalent dose (mg/kg/day); mean ± SD	0.12	–	–
Time between diagnosis and complete remission, months, median (IQR)	5 (3–8)	5 (3–7)	5 (3–7)
Time between diagnosis and first relapse, months, median (IQR)	29 (18–44)	–	–
Disease‐free time, months, median (IQR)	22 (12–36)	–	–
Follow‐up time, months, median (IQR)	78 (60–103.3)	70 (50–91.5)	74 (58–98)
ELISA, U/mL; median (IQR)			
At diagnosis			
Anti‐Dsg1^a^	62.9 (10.6–151.0)	30.3 (11.1–109.3)	56.8 (10.6–121.1)
Anti‐Dsg3^a^	142.8 (10.7–176.7)	137.8 (29.5–180.1)	139.6 (14.4–180.1)
At remission			
Anti‐Dsg1^b^	8.7 (5.8–26.6)	8.1 (6.2–10.6)	8.4 (5.8–12.5)
Anti‐Dsg3^b^	7.3 (4.3–94.7)	5.8 (4.2–69.6)	6.8 (4.2–93.2)
At relapse			
Anti‐Dsg1^c^	19.1 (8.0–102.1)	–	–
Anti‐Dsg3^c^	83.9 (4.4–156.1)	–	–
Serological subtypes, *n* (%)			
Anti‐Dsg1‐positive/anti‐Dsg3‐positive	35 (38.9)	24 (45.3)	59 (41.3)
Anti‐Dsg1‐positive/anti‐Dsg3‐negative	25 (27.8)	12 (22.6)	37 (25.9)
Anti‐Dsg1‐negative/anti‐Dsg3‐positive	30 (33.3)	17 (32.1)	47 (32.9)

BSA, body surface area; cPV, cutaneous pemphigus vulgaris; Dsg, desmoglein; IQR, interquartile range; mcPV, mucocutaneous pemphigus vulgaris; mPV, mucosal pemphigus vulgaris; OSA, oral surface area; PF, pemphigus foliaceus; SD, standard deviation.

^a^ At diagnosis, 60 relapsing and 36 nonrelapsing patients had a positive anti‐Dsg1 autoantibody titre, and 65 and 41, respectively, had a positive anti‐Dsg3 autoantibody titre;

^b^ at clinical remission, 25 relapsing and 8 nonrelapsing patients had a positive anti‐Dsg1 autoantibody titre, and 43 and 19 had a positive anti‐Dsg3 autoantibody titre;

^c^ at relapse, 45 relapsing patients had positive anti‐Dsg1 autoantibody titres and 53 had positive anti‐Dsg3 autoantibody titres.

Skin involvement was observed in 117 (81.8%) of the 143 patients, with BS of 1 in 27 patients (18.9%), BSA of 2 in 62 (43.4%) and BSA of 3 in 28 (19.6%) patients, while oral involvement was observed in 95 patients (66.4%), with OSA of 1 in 17 patients (11.9%), OSA of 2 in 58 (40.6%) and OSA of 3 in 20 (14.0%). Median antibody titre was 56.8 U/mL (IQR 10.6–121.1 U/mL) for anti‐Dsg1 antibodies and 139.6 U/mL (IQR 14.4–180.1 U/mL) for anti‐Dsg3. Of the 143 patients, 37 (25.9%) had exclusively anti‐Dsg1 and 47 (32.9%) had exclusively anti‐Dsg3 autoantibody positivity at diagnosis, while (41.3%) patients had both anti‐Dsg1 and anti‐Dsg3 autoantibodies at diagnosis. Median time to remission was not significantly different between relapsing and nonrelapsing patients [5 months (IQR 3–8 months) vs. 5 months (IQR 3–7 months); *P* = 0.52]. At diagnosis, neither median anti‐Dsg1 or Dsg3 antibody values were significant between relapsing and nonrelapsing patients: 62.9 U/mL vs. 30.3 U/mL (*P* = 0.20) for Dsg1 and 142.8 U/mL vs. 137.8 U/mL in (*P* = 0.56) for Dsg3. Of all 143 patients at diagnosis, moderate yet statistically highly significant correlations between anti‐Dsg1 and BSA (Spearman ρ = 0.45, *P* < 0.001) and between anti‐Dsg3 and OSA (ρ = 0.53, *P* < 0.001) were found. At remission, median anti‐Dsg1 antibody values in relapsing and nonrelapsing patients were 8.7 U/mL vs. 8.1 U/mL (*P* = 0.46), respectively, while median anti‐Dsg3 antibody values were 7.3 U/mL vs. 5.8 U/mL (*P* = 0.49), respectively.

No subsequent serological negativity and positivity was recorded in those with positive and negative anti‐Dsg1 and/or anti‐Dsg3 antibody titre at remission in the 6 months following the achievement of remission.

Induction therapy at diagnosis with either systemic corticosteroid alone (*n* = 69; 48.3%) or combined with immunosuppressive adjuvant drugs (*n* = 74; 51.7%) was documented. Maintenance therapy at first relapse consisted mainly of systemic corticosteroid monotherapy, with (*n* = 36; 40.0%) or without (*n* = 42; 46.7%) an immunosuppressive adjuvant drug. Mean systemic corticosteroid dosage at the time of relapse was prednisone 0.12 mg/kg/day. Only one patient was taking a nonsteroidal immunosuppressive drug, and 11 patients (12.2%) were not taking any medication.

After stratification according to pemphigus subtype at diagnosis, no statistically significant differences in antibody titres of anti‐Dsg1 or anti‐Dsg3 at diagnosis and remission or in time to remission were found between relapsing and nonrelapsing patients.

### Clinical and immunopathological features of relapsing patients

In relapsing patients, median time to remission was 5 months (IQR 3–8 months) and median time to relapse was 29 months (IQR 18–44 months) with a median disease‐free interval of 22 months (IQR 12–36 months).

The median value of anti‐Dsg1 antibodies was 62.9 U/mL at diagnosis, decreasing to 8.7 U/mL at remission and subsequently increased to 19.1 U/mL at first relapse. The median reduction in within‐patient anti‐Dsg1 titres at relapse compared with diagnosis (i.e. the median of the difference between titre at diagnosis and titre at relapse for each patient) was 8.3 U/mL (*P* = 0.001).

The median value of anti‐Dsg3 antibodies was 142.8 U/mL at diagnosis, decreasing to 7.3 U/mL at remission and subsequently increasing to 83.9 U/mL at first relapse. The median reduction in within‐patient anti‐Dsg3 titres at relapse compared with diagnosis was 2.7 U/mL (*P* = 0.06). At relapse, significant correlations between anti‐Dsg1 and BSA (ρ = 0.45, *P* < 0.001) and between anti‐Dsg3 and OSA (ρ = 0.41, *P* < 0.001) were noted.

### Clinical, demographic and serological predictors of relapse

In the total patient cohort, a BSA value of 3 predicted a higher risk of relapse compared with BSA < 3 (OR = 3.30, 95% CI 1.17–9.28; *P* = 0.02), as did a positive titre of either anti‐Dsg1 or anti‐Dsg3 autoantibodies at remission compared with being both negative (OR = 2.42, 95% CI 1.21–4.85, *P* = 0.01).

Considering anti‐Dsg1 and anti‐Dsg3 autoantibody positivity separately, both at diagnosis and at remission, neither was found to be a statistically significant predictor of relapse. However, a higher, although not statistically significant, risk of relapse (OR = 2.16, 95% CI 0.90–5.23, *P* = 0.09) was noted for patients with positive anti‐Dsg1 autoantibodies at remission.

Concerning induction therapy at diagnosis across our cohort, use of systemic corticosteroids plus immunosuppressive adjuvant drugs vs. systemic corticosteroid monotherapy predicted a higher, yet not statistically significant, risk of relapse (OR = 1.51, 95% CI 0.76–2.99, *P* = 0.24).

Considering patients with exclusively anti‐Dsg3 autoantibody positivity (i.e. those with positive anti‐Dsg3 and negative anti‐Dsg1 autoantibodies) at diagnosis (*n* = 47), failure to achieve anti‐Dsg3 titre negativity at clinical remission was a statistically significant predictor of relapse (OR = 7.89, 95% CI 2.06–30.21, *P* < 0.01). Conversely, in patients with exclusively anti‐Dsg1 autoantibody positivity (i.e. those with positive anti‐Dsg1 and negative anti‐Dsg3 autoantibodies) at diagnosis (*n* = 37), failure to achieve anti‐Dsg1 titre negativity at clinical remission was not a reliable predictor of relapse (OR = 1.08, 95% CI 0.27–4.29; *P* = 0.91). Finally, failure to achieve anti‐Dsg1 negativity at clinical remission in patients with both anti‐Dsg1 and anti‐Dsg3 autoantibody positivity at diagnosis (*n* = 59) was a statistically significant predictor of relapse (OR = 5.74, 95% CI 1.15–28.61; *P* = 0.03) (Table 2).

**Table 2:** Predictors of relapse according to clinical and immunopathological parameters in patients with pemphigus in different serological subsets at diagnosis.

Parameter	Serological subtypes						All patients (*n* = 143)	
	Anti‐Dsg1 positive/anti‐Dsg3 negative (*n* = 37)		Anti‐Dsg1 negative/anti‐Dsg3 positive (*n* = 47)		Anti‐Dsg1 positive/anti‐Dsg3 positive (*n* = 59)			
	OR (95% CI)	*P*	OR (95% CI)	*P*	OR (95% CI)	*P*	OR (95% CI)	*P*
Age, years								
≤ 45	1^a^	0.69	1^a^	0.74	1^a^	0.10	1^a^	0.17
46–60	0.47 (0.07–3.34)		0.92 (0.20–4.31)		0.22 (0.05–0.88)		0.43 (0.18–1.04)	
> 60	0.80 (0.12–5.41)		0.59 (0.15–2.36)		0.42 (0.11–1.57)		0.58 (0.25–1.35)	
Sex								
M	1^a^	0.57	1^a^	0.98	1^a^	0.33	1^a^	0.33
F	1.52 (0.37–6.30)		1.02 (0.31–3.36)		1.69 (0.59–4.85)		1.40 (0.71–2.79)	
Pemphigus subtype								
mcPV	1^a^	0.86	1^a^	0.98	1^a^	0.18	1^a^	0.52
cPV	^c^		–^c^		0.63 (0.15–2.68)		1.51 (0.46–4.88)	
mPV	1.00 (0.03–29.81)		1.15 (0.32–4.08)		6.26 (0.72–54.75)		1.80 (0.69–4.64)	
PF	2.22 (0.27–18.37)		–^c^		–^c^		1.68 (0.67–4.19)	
Tissue involvement								
Mucosal	1^a^	0.62	1^a^	0.98	1^a^	0.18	1^a^	0.32
Cutaneous	2.44 (0.14–43.47)		^c^		0.10 (0.01–1.17)		0.90 (0.32–2.56)	
Mucocutaneous	1.01 (0.03–29.81)		0.87 (0.25–3.11)		0.16 (0.02–1.40)		0.56 (0.22–1.44)	
BSA								
0–2	1^a^	0.08	–^b^		1^a^	0.15	1^a^	0.02
3	4.62 (0.84–25.49)		–^b^		2.80 (0.68–11.52)		3.30 (1.17–9.38)	
OSA								
0–2	–^b^		1^a^	0.85	1^a^	0.41	1^a^	0.48
3	–^b^		0.83 (0.13–5.56)		1.73 (0.47–6.44)		1.44 (0.52–4.01)	
Body site involvement								
Nasal and laryngeal								
Yes	–		1^a^	0.88	1^a^	0.85	1^a^	0.98
No	–^b^		0.87 (0.14–5.31)		0.89 (0.25–3.14)		0.99 (0.36–2.69)	
Anogenital								
Yes	–^b^		1^a^	0.47	1^a^	0.49	1^a^	0.98
No	–^b^		1.67 (0.42–6.59)		0.55 (0.10–3.08)		0.99 (0.36–2.69)	
Ocular								
Yes	–^b^		1^a^	0.55	1^a^	0.35	1^a^	0.81
No	–^b^		1.87 (0.24–14.61)		0.34 (0.04–3.22)		0.84 (0.20–3.51)	
Therapy at diagnosis								
Systemic CS monotherapy	1^a^	0.42	1^a^	0.68	1^a^	0.39	1^a^	0.24
Systemic CS plus immunosuppressive adjuvant therapy	1.78 (0.44–7.18)		1.29 (0.39–4.24)		1.58 (0.55–4.48)		1.51 (0.76–2.99)	
Antibody positivity at diagnosis								
Anti‐Dsg1								
Yes	NA		NA		NA		1^a^	0.88
No	NA						1.06 (0.51–2.19)	
Anti‐Dsg3								
Yes	NA		NA		NA		1^a^	< 0.50
No	NA						1.31 (0.60–2.90)	
Antibody negativity at remission								
Anti‐Dsg1								
Yes	1^a^	0.91	NA		1^a^	0.03	1^a^	0.09
No	1.08 (0.27–4.29)				5.74 (1.15–28.61)		2.16 (0.90–5.23)	
Anti‐Dsg3								
Yes	NA		1^a^	< 0.01	1^a^	0.82	1^a^	0.11
No			7.89 (2.06–30.21)		1.13 (0.40–3.21)		1.78 (0.88–3.59)	
Both anti‐Dsg1 and anti‐Dsg3^d^								
Yes	–^b^		^b^		^b^		1^a^	0.01
No	–^b^						2.42 (1.21–4.85)	

BSA, body surface area; cPV, cutaneous pemphigus vulgaris; CS, corticosteroid; Dsg, desmoglein; mcPV, mucocutaneous pemphigus vulgaris; mPV, mucosal pemphigus vulgaris; NA, not applicable; OSA, oral surface area. Multivariate analysis estimates for the ‘All patients’ group: Anti‐Dsg1 and anti‐Dsg3 negativity at remission (No vs. Yes): OR = 2.39 (1.17–4.85), *P* = 0.02; BSA (3 vs. 0–2), OR = 3.24 (1.13–9.27), *P* = 0.029.

^a^ Reference category;

^b^ model fit was not possible because data were too sparse;

^c^ OR estimates were not possible because data were too sparse;

^d^ parameter was considered only in the analysis performed on all 143 patients.

## Discussion

In the past few decades, several studies have investigated possible clinical and/or laboratory predictors of relapse in pemphigus, focusing mainly on the relationship between disease activity and autoantibody titres evaluated by means of ELISA. Given that pemphigus relapses usually occur in the first 2 years after disease onset, only patients with at least a 3‐year follow‐up were included in this study. In line with the work by Kyriakis and Tosca,^2^ median time from diagnosis to first relapse was 29 months, with a median disease‐free interval of about 22 months.

Among the collected clinical variables, only a BSA score of ≥ 3 at diagnosis, corresponding to ≥ 15% BSA involvement, was shown to be a significant predictor of relapse compared with less extensive cutaneous disease scored as BSA < 3. In contrast to previous studies^4^, ^5^, ^25^ that indicated possible role of mucosal involvement at pemphigus onset as a predictor of relapse, we found that neither mucosal involvement at onset nor any of the other clinical variables were significant predictive factors of relapse in our cohort.

Several investigations^9^, ^10^, ^11^, ^12^, ^13^, ^14^, ^15^, ^16^ have validated the correlation between disease activity and anti‐Dsg1 and/or anti‐Dsg3 autoantibody titres in pemphigus. In agreement with these, we noted in our cohort a significant correlation at both diagnosis and relapse between anti‐Dsg1 autoantibody titres and BSA scores, and between anti‐Dsg3 autoantibody titres and OSA scores. The role of autoantibody titres as immunological predictors of relapse is more nuanced, with the available evidence being controversial.^9^, ^14^, ^19^

In our study, three serological subsets were devised to assess the utility of each autoantibody titre within highly coherent subgroups. When our cohort was assessed in its entirety, neither anti‐Dsg1 nor anti‐Dsg3 autoantibody positivity, at either diagnosis or remission, was found to be a significant predictor of relapse. However, focusing separately on each serological subset, we found that failure to achieve anti‐Dsg1 autoantibody titre negativity predicted relapse in patients who are anti‐Dsg1‐ and anti‐Dsg3‐positive, while failure to achieve anti‐Dsg3 titre negativity at clinical remission was a valuable predictor of relapse in patients with exclusively anti‐Dsg3 autoantibody positivity at diagnosis. By contrast, anti‐Dsg1 autoantibody titre did not provide adequate immunological guidance in predicting relapse in patients who are anti‐Dsg1‐positive and anti‐Dsg3‐negative. It could be speculated that the aforementioned serological subsets at diagnosis faithfully identify groups of patients with homogeneous immunopathological profiles, and it is conceivable that monitoring such groups might allow a better prediction of relapse than considering anti‐Dsg1 or anti‐Dsg3 separately. Failure of anti‐Dsg1 antibodies in predicting relapse in the subset with exclusively anti‐Dsg1 positivity might be due to the high proportion of patients with elevated nonpathogenetic anti‐Dsg1 at remission who did not relapse, as also suggested by Kamiya *et al*.^26^

Our study has some limitations. Owing to its retrospective nature, assessment of disease severity was not performed by means of a validated scoring system, such as the Pemphigus Disease Area Index or Autoimmune Bullous Skin Disorder Intensity Score. Moreover, although a minimum of 36 months of follow‐up was required for inclusion, the acual duration of follow‐up varied. However, considering that most pemphigus relapses occur within the first 2 years from onset, it is likely that patients classified as ‘nonrelapsing’ in our cohort did not relapse after cessation of follow‐up. Further, to simplify our analyses, we focused on clinical and serological data at first relapse only. Concerning induction therapy at diagnosis, the use of an immunosuppressive adjuvant drug alongside systemic corticosteroids did not predict a lower relapse risk relative to systemic corticosteroid monotherapy; in fact, the opposite was true, albeit not reaching statistical significance. This finding possibly reflects the choice of immunosuppressive adjuvants in patients with a more severe clinical picture at diagnosis. It also shows the limited impact of induction therapy at diagnosis as a confounder in the rest of our analyses. It was not feasible to retrospectively assess the predictive role of treatment during follow‐up, as this would have required an arbitrary timepoint selection for the comparison of nonrelapsers vs. relapsers (i.e. treatment at an arbitrary timepoint during remission vs. treatment at first relapse, respectively). However, it must be emphasized that most patients were receiving systemic corticosteroids and/or adjuvant treatments at the moment of relapse, with only a minority being untreated. Finally, the small sample sizes of individual subgroups stratified by clinical subtype lacked the statistical power needed to demonstrate an effect in relapse prediction.

## Conclusion

Patient classification based on anti‐Dsg1 and anti‐Dsg3 autoantibody positivity at diagnosis, regardless of pemphigus subtype and subsequent assessment of antibody titres at clinical remission, may be of use in predicting relapses. More specifically, failure to achieve anti‐Dsg3 titre negativity at clinical remission appears to be a significant predictor of relapse in patients with isolated anti‐Dsg3 autoantibody positivity at diagnosis, whereas failure to achieve anti‐Dsg1 titre negativity at remission seems to predict relapse only in patients with both anti‐Dsg1 and anti‐Dsg3 autoantibody positivity, but not in those who had isolated anti‐Dsg1 autoantibody positivity at diagnosis.

## Acknowledgement

Open Access Funding provided by Universita degli Studi di Milano within the CRUI‐CARE Agreement. [Correction added on 18 May 2022, after first online publication: CRUI funding statement has been added.]

**What's already known about this topic?**

- Clinical and immunopathological factors predictive of relapse and earliness of relapse in pemphigus have not yet been clearly identified.
- Although anti‐Dsg1 and anti‐Dsg3 titres have been directly correlated to disease activity, the role of serological monitoring through serial ELISAs in the follow‐up of patients with pemphigus is controversial.

**What does this study add?**

- A BSA score of 3 is a significant predictor of relapse compared with a BSA score of < 3.
- Autoantibody titre negativity in clinical remission in patients stratified on the basis of their anti‐Dsg1 and anti‐Dsg3 profile at diagnosis may be a reliable tool in predicting relapse.

## Supporting information


**Table S1.** Characteristics of patients with relapsing pemphigus stratified based on pemphigus subtype.
