# Supplementary material for: Clinical and serological predictors of relapse in pemphigus: a study of 143 patients
Source: Clin Exp Dermatol. 2021 Aug 27;47(1):98–106. doi: 10.1111/ced.14854 (PMC9290045; doi:10.1111/ced.14854)
Supplement: Supplementary file 1 — Table S1. Characteristics of patients with relapsing pemphigus stratified based on pemphigus subtype. [file CED-47-98-s001.docx]

**Table S1.** Characteristics of patients with relapsing pemphigus stratified based on pemphigus subtype

|  | | **PF (n=20)** | **cPV (n=10)** | **mcPV (n=41)** | **mPV (n=19)** |
| --- | --- | --- | --- | --- | --- |
| **Time from diagnosis to clinical remission, months, median (IQR)** | | 6.0 (3.0-8.5) | 6.5 (4.0-10.0) | 5.0 (3.0-8.0) | 4.0 (3.0-6.0) |
| **Time from diagnosis to first relapse, months, median (IQR)** | | 33.0 (18.0-45.0) | 33.0 (20.0-60.0) | 28.0 (20.0-44.0) | 18.0 (12.0-36.0) |
| **Disease-free interval, months, median (IQR)** | | 21.0 (14.5-36.0) | 30.0 (16.0-55.0) | 23.0 (12.0-36.0) | 15.0 (7.0-30.0) |
| **BSA at first relapse, n (%)** | 0 | 0 | 1 (10) | 14 (34.2) | 13 (68.4) |
|  | 1 | 8 (40) | 2 (20) | 11 (26.8) | 3 (15.8) |
|  | 2 | 10 (50) | 6 (60) | 15 (36.6) | 2 (10.5) |
|  | 3 | 2 (10) | 1 (10) | 1 (2.4) | 1 (5.3) |
| **OSA at first relapse, n (%)** | 0 | 20 (100) | 8 (80) | 18 (43.9) | 6 (31.6) |
|  | 1 | 0 | 1 (10) | 8 (19.5) | 4 (21.1) |
|  | 2 | 0 | 1 (10) | 11 (26.8) | 9 (47.4) |
|  | 3 | 0 | 0 | 4 (9.8) | 0 |
| **Involved mucosal sites at first relapse, n (%)** | Oral mucosa | 0 | 2 (20) | 23 (56.1) | 13 (68.4) |
|  | Nasal/laryngeal mucosa | 0 | 1 (10) | 4 (9.8) | 4 (21.1) |
|  | Anogenital mucosa | 0 | 1 (10) | 4 (9.8) | 0 |
|  | Conjunctiva | 0 | 0 | 2 (4.9) | 0 |
| **ELISA at diagnosis, median value, U/ml (IQR)** | anti-Dsg1^§^ | 147.0 (75.7-189.8) | 67.1 (11.3-175.9) | 41.3 (9.3-117.9) | 18.1 (9.1-74.4) |
|  | anti-Dsg3^§^ | 4.1 (3.3-5.3) | 95.0 (28.1-142.8) | 170.4 (135.0-186.8) | 160.1 (122.9-188.6) |
| **ELISA at remission, median value, U/ml (IQR)** | anti-Dsg1^§§^ | 33.0 (5.4-69.9) | 10.2 (6.4-24.8) | 8.4 (5.8-10.6) | 7.3 (5.3-9.1) |
|  | anti-Dsg3^§§^ | 4.7 (2.7-5.5) | 6.0 (3.5-88.9) | 57.2 (6.3-119.1) | 69.8 (6.6-162.5) |
| **ELISA at relapse, median value, U/ml (IQR)** | anti-Dsg1^§§§^ | 100.0 (60.9-150.9) | 85.2 (7.9-102.1) | 9.3 (8.0-60.7) | 9.2 (7.8-85.5) |
|  | anti-Dsg3^§§§^ | 4.3 (3.0-4.8) | 99.05 (54.6-150.4) | 137.5 (6.7-164.1) | 117.9 (56.1-176.3) |
| **Median duration of the first relapse, months (IQR)** | | 6.0 (4.0-9.5) | 5.5 (2.8-11.5) | 4.0 (3.0-7.0) | 5.0 (4.0-6.0) |

BSA=body surface area; cPV= cutaneous pemphigus vulgaris; Dsg=desmoglein; ELISA=enzyme linked immunosorbent assay; IQR= interquartile range; mcPV= mucocutaneous pemphigus vulgaris; mPV= mucosal pemphigus vulgaris; OSA=oral surface area

§ At diagnosis, among relapsers, 20 patients with PF, 6 with cPV, 25 with mcPV and 9 with mPV had a positive anti-Dsg1 titer; while 0 patients with PF, 8 with cPV, 39 with mcPV and 18 with mPV had a positive anti-Dsg3 titer.

§§ At remission, among relapsers, 11 patients with PF, 4 with cPV, 8 with mcPV and 2 with mPV had a positive anti-Dsg1 titer; while 0 patients with PF, 3 with cPV, 26 with mcPV and 14 with mPV had a positive anti-Dsg3 titer.

§§§ At relapse, 16 patients with PF, 6 with cPV, 18 mcPV and 5 with mPV had a positive anti-Dsg1 titer; while 0 patients with PF, 8 with cPV, 30 with mcPV and 15 with mPV had a positive anti-Dsg3 titer.
